# Genotyping of *ABCC8*, *KCNJ11,* and *HADH* in Iranian Infants with Congenital Hyperinsulinism

**DOI:** 10.1155/2021/8826174

**Published:** 2021-05-13

**Authors:** Somayyeh Hashemian, Reza Jafarzadeh Esfehani, Siroos Karimdadi, Nosrat Ghaemi, Peyman Eshraghi, Najmeh Malekzadeh Gonabadi, Amirhossein Sahebkar, Rahim Vakili, Mohammad Reza Abbaszadegan

**Affiliations:** ^1^Department of Pediatric Diseases, Akbar Hospital, Faculty of Medicine, Mashhad University of Medical Sciences, Mashhad, Iran; ^2^Department of Medical Genetics, Faculty of Medicine, Mashhad University of Medical Sciences, Mashhad, Iran; ^3^Faculty of Sciences, Sistan and Baluchestan University, Zahedan, Iran; ^4^Biotechnology Research Center, Pharmaceutical Technology Institute, Mashhad University of Medical Sciences, Mashhad, Iran; ^5^Applied Biomedical Research Center, Mashhad University of Medical Sciences, Mashhad, Iran; ^6^School of Pharmacy, Mashhad University of Medical Sciences, Mashhad, Iran; ^7^Medical Genetic Research Center, Medical School, Mashhad University of Medical Sciences, Mashhad, Iran

## Abstract

**Background:**

Congenital hyperinsulinism (CHI) is a heterogeneous disease with various underlying genetic causes. Among different genes considered effective in the development of CHI, *ABCC8*, *KCNJ11,* and *HADH* genes are among the important genes, especially in a population with a considerable rate of consanguineous marriage. Mutational analysis of these genes guides clinicians to better treatment and prediction of prognosis for this rare disease. The present study aimed to evaluate genetic variants in *ABCC8*, *KCNJ11,* and *HADH* genes as causative genes for CHI in the Iranian population.

**Methods:**

The present case series took place in Mashhad, Iran, within 11 years. Every child who had a clinical phenotype and confirmatory biochemical tests of CHI enrolled in this study. Variants in *ABCC8*, *KCNJ11,* and *HADH* genes were analyzed by the polymerase chain reaction and sequencing in our patients.

**Results:**

Among 20 pediatric patients, 16 of them had variants in *ABCC8*, *KCNJ11,* and *HADH* genes. The mean age of genetic diagnosis was 18.6 days. A homozygous missense (c.2041-21G > *A*) mutation in the *ABCC8* gene was seen in three infants. Other common variants were frameshift variants (c.3438dup) in the *ABCC8* gene and a missense variant (c.287-288delinsTG) in the *KCNJ11* gene. Most of the variants in our population were still categorized as variants of unknown significance and only 7 pathogenic variants were present.

**Conclusion:**

Most variants were located in the *ABCC8* gene in our population. Because most of the variants in our population are not previously reported, performing further functional studies is warranted.

## 1. Introduction

Congenital hyperinsulinism (CHI) is an important cause of hypoglycemia during the neonatal period and infancy. While transient hypoglycemia could be considered as a common metabolic abnormality among neonates, only 1% of these infants develop repetitive or persistent episodes of hypoglycemia [1]. During CHI, an increased level of insulin within the infant's blood decreases free glucose and results in neurological dysfunction. Repeated episodes of hypoglycemia can also result in neurological damage including mental retardation and developmental delay [2, 3]. Considering the heterogeneous nature of CHI, not all affected infants will have the same clinical presentation and histological findings.

CHI diagnostic algorithms start from the metabolic evaluation followed by molecular investigation and genotyping. Although genetic analysis is the most useful test in hyperinsulinism diagnostic approach, specific radiological modalities such as 18-F-DOPA positron emission tomography (PET) scan are also used to specify lesions [4]. Variants in some specific genes that regulate insulin secretion are considered to be responsible for the development of CHI. *ABCC8* and *KCNJ11* are among the most common genes involved in CHI [5, 6]. These genes encode subunits of ATP sensitive K^+^ (KATP) channels in beta cells. *ABCC8* and *KCNJ11* gene variants impair the function of these channels resulting in disrupted membrane depolarization and dysregulated entry of calcium ion ending up in insulin release into plasma [2, 7]. Both of these genes are located on the chromosome 11p15.1 and separated by a few kilobases. *HADH* is the other well-known causative gene for CHI and variants in this gene cause enzymatic defects. *HADH* gene-inactivating variants have been described in CHI patients, resulting in decreased enzymatic activity of L-3-hydroxyacyl-CoA-dehydrogenase [8, 9]. Different types of variants including missense, nonsense, frameshift, and insertions or deletions inactivate these three genes. Determination of such variants in candidate genes is useful in various ways including choosing the best treatment approach [5]. Diazoxide is a KATP channel agonist and the first line of therapy in most of the CHI patients. Those patients who are not responsive to diazoxide may use octreotide as an alternative treatment. Surgical therapy might be used for those CHI patients who are not responsive to pharmacological therapies. Recessive variants in *ABCC8* and *KCNJ11* genes are usually considered to be less responsive to some therapies including diazoxide while compound heterozygotes may have better treatment responses [5, 10]. In contrast to recessive variants, dominant mutations will have a better treatment response [5]. Many case reports and case series have reported new variants in *ABCC8* and *KCNJ11* genes in different populations. We have previously demonstrated rare genetic variants in these two genes in an Iranian population and also demonstrated the clinical efficacy of sirolimus therapy in CHI patients [11]. The prevalence of specific variants in each population seems to be unique and genotyping for CHI has not been widely reported in the Iranian population. Other alternative diagnostic options for CHI include PET scan that helps identifying localization of focal lesions. In the present report, we explored the variants in *ABCC8* and *KCNJ11* genes as the most common genes involved in CHI. Hence, we aimed to evaluate value of genotyping in the diagnostic process, which is a crucial step in CHI treatment.

## 2. Material and Methods

### 2.1. Subjects

The present case series was approved by the Mashhad University of Medical Sciences Ethics Committee and included pediatric patients who were referred to the pediatric ward of the Imam Reza Hospital (Mashhad, Iran) from 2008 to 2017. Every neonate/child who had clinical and laboratory features of persistent hypoglycemia underwent assessment of “critical” sample in hypoglycemic state (with hypoglycemia defined as plasma glucose less than 50 mg/dL). Cases who had (1) hyperinsulinemia (defined as plasma insulin greater than 2 *μ*U/mL), or (2) hypofatty acidemia (plasma free fatty acids less than 1.5 mmol/L), or (3) hypoketonemia (defined as plasma beta-hydroxybutyrate less than 2 mmol/L), or (4) inappropriate glycemic response to 1 mg intravenous glucagon (changes in plasma glucose greater than 40 mg/dL) were defined to have hyperinsulinism, and treatment with dextrose fluid therapy, diazoxide, and then octreotide were administered if needed [10]. All patients who had confirmed diagnosis of hyperinsulinism underwent genetic evaluation and were enrolled in the present study if their parents filled an informed consent form. Patients underwent molecular diagnosis for hyperinsulinemic hypoglycemia after genetic counseling. Because of the limitations, genetic testing was limited to the most common genes involved in CHI. Moreover, PET-CT data and surgical specimens of all patients were not available. Among the study population, only six patients were previously reported in the literature and their genotypes and clinical data are considered in the present study as well. Some of them had closed follow-up with PET scan study or surgery, which has been reported previousely (Table 1) [11].

### 2.2. Sampling and DNA Extraction

To perform molecular tests, venous blood (3 mL) was taken from all subjects and their parents in EDTA tubes. Genomic DNA was extracted using the salting-out method. DNA samples' quality and quantity were examined using nano-drop and gel-electrophoresis and stored in the freezer at −70°C.

### 2.3. PCR Sequencing

Mutational analysis of coding and flanking intronic regions of the *KCNJ11, HADH,* and *ABCC8* genes was performed using Sanger sequencing following PCR fragment amplification. Interpretation of the pathogenicity of the genetic variants in these genes was checked by VarSome software based on the American College of Medical Genetics (ACMG) instructions [12, 13]. Molecular genetic analysis was performed in the genetic lab of the Mashad University of Medical Sciences and/or genetic lab of the Exeter University in UK.

## 3. Results

Twenty patients were enrolled in the present study. Among this population, four infants did not have genetic variants in *ABCC8*, *KCNJ11,* or *HADH* gene. Among the other 16 infants, 11 patients were female and five were male. The mean age of genetic diagnosis was 18.6 days in those who had *ABCC8* or *KCNJ11* variants (except one case who had delay in the diagnosis of CHI). Most of the infants had their genetic tests few weeks after birth. The patient with the most delayed genetic diagnosis was the one who was diagnosed at the age of 3.5 years. Most of the patients had variants in the *ABCC8* gene (Table 1). A missense and aberrant splicing variants in the *ABCC8* gene (c.2041-21G > *A*) were seen in three infants. Other common variants were frameshift variants (c.3438dup) in the *ABCC8* gene and a missense variant (c.287-288delinsTG) in the *KCNJ11* gene. According to the ACMG guideline, most of the genetic variants in our population were reported as variants with uncertain significance.

## 4. Discussion

According to the results of this study, more than half of the study population with a clinical diagnosis of CHI had variants in the *ABCC8* gene. Additionally, most of these patients had variants with uncertain significance in *ABCC8* or *KCNJ11* genes.

Recurrent or persistent hypoglycemia is the first clue for the diagnosis of CHI. The presentation of CHI usually begins during the neonatal period and some types of CHI including focal CHI can present later in life. Less than half of the children with CHI have the genetic form of this disease [7]. At least variants in nine genes have been reported to be related to CHI. Among these genes, *ABCC8* and *KCNJ11* carry most of the mutations that cause CHI and other mutations are common in specific populations. HADH gene is among the other genes that have been reported to be common in countries with considerable consanguinity including Iran [14]. Variants in both *KCNJ11* and *ABCC8* genes, which are located on chromosome 11p15.1, are characterized by defective Kir6.2 and SUR1 subunits of ATP-dependent K channels in B-cells [6]. KATP channel variants result in various degrees of dysfunction ranging from partial to complete loss of channel function in B-cells [15]. Recessive and dominant variants in both of these genes can cause CHI [7].

Many studies from around the world have evaluated the heterogeneous genotypes of CHI patients. It is noteworthy to mention that even in the same country, a wide range of variants in *ABCC8* and *KCNJ11* genes are present. As summarized in Table 1, only a small number of variants have been previously reported in the literature. Only a few studies from Iran evaluated the genotype of CHI patients. Recently, a study from Iran evaluated six patients with CHI and the only similar variants to our study were the c.96 C *G* variants in the *ABCC8* gene [14]. Alaei et al. [14] reported that one of their patients had this variant in homozygous form while our patient had this variant in compound heterozygous form. Another study from Iran evaluated 23 patients with hyperinsulinemic hypoglycemia [16]. The authors demonstrated that after screening for *ABCC8, HADH, HNF4A, KCNJ11*, *GLUD1*, and *GCK*, only 4 patients did not show any variant in these genes. Most of their patients had variants in the *HADH* gene and the *ABCC8* gene was the next gene with frequent variants. The only similar variants between this study and the present one are the splicing variants in the *ABCC8* gene (c.2041-21G > *A*) [16]. They reported that their female heterozygous patient had died at the age of 3 months. In our population, we reported a compound heterozygous male with this variant and three homozygous infants. The c.2041-21G > *A* variants were the most common variants in our population.

Other genetic variants were slightly similar to studies from other countries. Kapoor et al. demonstrated that among a CHI population who were referred to a children hospital in England, c.331 G > *A* variant in the *ABCC8* gene was one of the most common variants [2]. Another study from England provided the same results. Among their 53 CHI patients in a 13-year period, only the c.331 G > *A* variant was similar to our study [17]. The c.331 G > *A* variants in *ABCC8* gene were also the only similar variants between our study and a retrospective Chinese study [18].

In our studied population, only one compound heterozygous variant was found. Compound heterozygous status has been reported in other studies while Senniappan et al. have previously reported the same heterozygous variants in the Iranian population [16, 19]. Their patient was a female child who died at 3 months of age [16]. Similar to the Senniappan et al.'s study, Mohnike et al. also demonstrated a case of paternally heterozygous child with c.2041-21G > *A* variant in the *ABCC8* gene and focal CHI [20]. Their patient has undergone surgery but the outcome was not mentioned [20]. Sousa-Santos et al. reported that even patients with the same compound heterozygous variants in the *ABCC8* gene (c.3576delG and c.742 C *T*) can have different phenotypes [21]. Moreover, a combination of heterozygous variants in *ABCC8* and *KCNJ11* genes has been reported to be responsible for CHI [22].

Although genetic analysis can be applied as an important tool for diagnostic and management purposes in some patients, genotyping in our population showed that most of the variants were still categorized as variants of unknown significance (VUS) in *ABCC8* or *KCNJ11* genes. However, further genetic studies are needed to unravel molecular basis of CHI.

### 4.1. Limitations

In the present study, we used Sanger sequencing for the genotyping of all patients, and therefore, some other possible genetic variants including copy number variations might have been undetectable by using this technique. Moreover, all of our patients did not undergo other diagnostic workups including PET scan. Only six patients with closed follow-up have been reported previously [11]. The high cost of these imaging and other molecular techniques including using multiplex ligation-dependent probe amplification or gene panel analysis by next generation sequencing might have limited the interpretation of the results of our research in its present form. Furthermore, we did not have histological specimens of our patients. Finally, we evaluated a particular gene panel as causative genes for CHI in the Iranian population and we were not able to investigate other genes.

## 5. Conclusion

The present study evaluated genetic variants in *ABCC8*, *KCNJ11,* and *HADH* genes in 20 children. Approximately, 75% of children with CHI phenotype had variants in *ABCC8* and *KCNJ11* genes. According to the present results, c.2041-21G > *A* missense variant in the *ABCC8* gene was the most common variant in our Iranian population. Most of the variants in our population were variants with unknown significance and only seven patients had pathogenic variants.

## Figures and Tables

**Table 1:** Genotypes and pathogenicity prediction by using online prediction tools and the clinical significance according to Varsome.

Gene	Gender	Birth weight (gr)	Symptom onset	BS-INS at diagnosis	molecular diagnosis	Variant	Zygosity	GnomAD allele frequency	Iranome allele frequency	Clinical significance (varsome.com)	Family history	Surgery	Response to Diazoxide	Clinical Comments (patients did not response to diazoxide underwent surgery or other drugs)
ABCC8	F	4200	4D	27–47	1M	c.331G>A	Hom^	0.000008	—	Pathogenic	—	50D	—	Received diazoxide and octreotide before surgery and sirolimus for 3 months after surgery #
						p.Gly111Arg								
	F	2600	1D	34–63	2.5M	c.2809C>T p.Leu937=	Hom^	—	—	Pathogenic	—	30D	—	Received diazoxide and sirolimus after surgery # histologic features: diffused islet hyperplasia
	F	4350	3D	22–53	1M	c.1671+1G>A	Hom^	—	—	Pathogenic	—	57D	—	—
	F	3400	3D	20–57	3M,6D	c.2041-21G>A	Hom^	0.00002	—	Likely Pathogenic	+∼	—	+	Received octreotide plus diazoxide in some weeks infancy,continued with diazoxide
	M	4000	2D	25–33	1M,4D						+∼	2M	—	PET scan: no uptake in pancreas He died because of sepsis following the surgery
	M	4200	1D	18–27	1M						+∼∼	—	—	Received diazoxide plus octreotide but lost follow up
	F	3100	2D	28–69	4M	c.3438dup p.Thr1147HisfsTer48	Het (pat)^∗^	—	—	Pathogenic	—	2M	—	—
	F	3000	4D	40–12	5M,22D	c.1109G>C p.Arg370Thr	Het (de novo)	—	—	Uncertain Significance	—	—	+	—
	F	2800	3D	25–38	6Y,9M	c.3438dup p.Thr1147HisfsTer48	Het (pat)^∗^	—	—	Pathogenic	—	55D	—	Received insulin after surgery because of hyperglycemia
	F	4200	1D	12–28	1M,2D	c.3151dup p.Cys1051LeufsTer64	Hom^	—	—	Pathogenic	—	—	—	Received diazoxide plus octreotide but lost follow up
	M	3150	4D	32–54	1M	c.96C>G p.Asn32Lys	Comp-Het	—	—	Uncertain Significance	—	—	—	Received sirolimus and responded to sirolimus therapy #
						c.2041-21G>A		0.00002		Uncertain Significance				
	M	2300	5D	44–34.6	3.5Y	c.3446G>A p.Cys1149Ty	Hom^	—	—	Likely Pathogenic	—	3.5Y	—	Received sirolimus plus diazoxide after surgery PET scan: avid lesion in the head of pancreas histologic features: islet cell hyperplasia
KCNJ11	F	3800	3D	28–44	1M,19D	c.287-288delinsTG p.Ala96Val	Hom^	—	—	Uncertain Significance	—	2M	—	—
	F	3500	2D	36–65	2M	c.370T>A p.Ser124Thr	Hom^	—	—	Uncertain Significance	—	2M	—	Received sirulimus ,octreotide and diazoxide before surgery. Continued with low doses of diazoxide after surgery # Pet Scan :a mild uptake in the head of pancreas
	F	4100	1D	28–28	22D	c.362T>G p.Phe121Cys	Hom^	—	—	Uncertain Significance	+∼∼	—	—	Did not responded to diazoxide alone , continued with octreotide and sirolimus #
HADH	M	3100	15D	38–14	1M	c.706C>T p.Arg236Ter	Hom^	0.00001	—	Pathogenic	—	—	+	PET scan: Uptake in the head of pancreas .Followed by pancreatectomy with no attacks of hypoglycemia

BS: Blood sugar (mg/dl); INS: insulin (IU/ml); D: day; M: Month; Y: year; Pat: paternal; Comp-Het: Compound heterozygote. # The patient previously reported from our center. ^ The mother and father are heterozygote for the variant. ^∗^ The mother did not have any variant in the three genes and the father was asymptomatic and carrying the variant. ∼ The patients are first cousins. ∼∼ The family had child with similar clinical manifestations but without genetic workups.

## Data Availability

Data are available from the corresponding author upon reasonable request.
